# The Phenology of *Aphalara itadori* in Canada: Timing of Spring Activity and the Onset of Oviposition

**DOI:** 10.3390/insects17040376

**Published:** 2026-04-01

**Authors:** Ian M. Jones, Sandy M. Smith, Robert S. Bourchier

**Affiliations:** Institute of Forestry & Conservation, University of Toronto, 33 Willcocks Street, Toronto, ON M5S 3B3, Canada

**Keywords:** *Aphalara itadori*, biological control, diapause, *Fallopia japonica*, oviposition, voltinism

## Abstract

The timing of spring activity and oviposition in the knotweed psyllid, *Aphalara itadori* Shinji (Hemiptera: Psyllidae), will affect the number of generations that this biological control agent can complete across its introduced range and its impact on invasive knotweed. We conducted a controlled overwintering experiment in Ontario, Canada, to observe patterns of *A. itadori* activity and oviposition in spring and to explore sources of spring mortality. *Aphalara itadori* were observed to be active in very early spring, with the first observation of a psyllid on a sentinel leaf occurring on 23 March 2021. The first viable eggs were laid between 18 and 20 April 2021, when mean maximum daily temperatures were ~14 °C and day length was 13.36 h. Psyllid activity/survival throughout the spring was not affected positively or negatively by access to foliage. Our results suggest that, in Canada, the timing of the first spring generations of *A. itadori* will likely be dictated by the phenology of the host plant rather than the biology of the insect. The results also suggest that spring mortality in *A. itadori*, from starvation or abiotic factors, is low even when knotweed emergence is late.

## Simple Summary

The timing of spring activity and oviposition in the knotweed psyllid, *Aphalara itadori* Shinji (Hemiptera: Psyllidae), will affect the number of generations that this biological control agent can complete across its introduced range and its impact on invasive knotweed. We conducted a controlled overwintering experiment in Ontario, Canada, to observe patterns of *A. itadori* activity and oviposition in spring. *Aphalara itadori* were observed to be active as early as 23 March, and the first viable eggs were laid between 18 and 20 April. Psyllid activity/survival throughout the spring was not affected positively or negatively by access to foliage. Our results suggest that, in Canada, the timing of the first spring generations of *A. itadori* will likely be dictated by the phenology of the host plant rather than the biology of the insect. The results also suggest that spring mortality in *A. itadori*, from starvation or abiotic factors, is low even when knotweed emergence is late.

## 1. Introduction

Predicting the number of generations that a weed biological control insect will have in any part of its introduced range is important because it affects the potential rate of population increase as well as the impact the agent may have on the target weed [1]. Additionally, understanding the thermal responses of biological control insects and their influence on voltinism is important for predicting any potential climatic mismatches that might occur in the introduced range [2]. The knotweed psyllid *Aphalara itadori* Shinji (Hemiptera: Psyllidae) has been released for the control of invasive knotweed both in Europe and North America. We set out to understand patterns of *A. itadori* activity in spring in Canada and to explore possible sources of spring mortality in the agent.

The knotweed species complex consists of three species—Japanese knotweed, *Fallopia japonica* (Houtt.) Ronse Decr. (Caryophyllales: Polygonaceae), giant knotweed, *F. sachalinensis* (F. Schmidt) Ronse Decraene, and Bohemian knotweed, *F.* x *bohemica* (Chrtek & Chrtková) J. P. Bailey—the latter of which is a hybrid of Japanese and giant knotweed [3]. All three plant species are herbaceous perennials that form tall, dense monoclonal stands [4,5], which limit native plant species through shading [6] and leaf-litter accumulation [7]. In addition to reducing native plant diversity, Japanese knotweed has been shown to negatively affect aquatic fauna by displacing plants on riverbanks that provide critical protection against soil erosion [8,9,10,11,12]. Perhaps the most economically significant threat of invasive knotweed species is damage to infrastructure. The species has highly vigorous root systems, capable of growing through concrete, and infestations frequently hinder development projects and affect property values [13]. Chemical and mechanical control of invasive knotweed is not feasible at the scale of the infestation. Even if above-ground foliage is successfully cleared, patches will rapidly regenerate from robust underground rhizomes [7,14,15]. Chemical control is also prohibited in many infestations due to their proximity to water bodies. Classical biological control represents a sustainable and scalable tool for the control of invasive knotweed at the landscape scale.

The knotweed psyllid, *A. itadori*, is host-specific to the invasive knotweed complex [16] and emerged in the early 2000s as the leading insect candidate for classical biological control [17]. Two biotypes of *A. itadori* were initially collected from Japan, one from Kyushu in 2004 (the southern biotype), which performs best on *F. japonica*, and the other from the northern Japanese island of Hokkaido in 2007 (the northern biotype), which performs best on *F. sachalinensis* [15,16]. A second population of the southern biotype was collected in 2016 by CABI UK. This line is thought to be better adapted to field conditions, having spent less time in captivity [18], and it was these psyllids (hereafter referred to as K2 psyllids) that were used in this study.

*Aphalara itadori* releases have been conducted in the United Kingdom (UK) in 2010, in Canada in late 2014, and most recently in the U.S. and the Netherlands in 2020. The largest releases, totaling over 200,000 individuals, have been conducted in British Columbia, Alberta, and Ontario, Canada (Bourchier, unpublished data). Across all of these release locations, diapausing adults released in the fall have been observed to overwinter successfully and lay large numbers of eggs in spring. The resulting populations have generally failed to persist for longer than two generations and have not reached densities capable of exerting any control over the weed [19]. In North America, nymphal survival appears to represent the biggest obstacle for the establishment of *A. itadori*, with both predation and foliage quality implicated as important mortality factors [20].

Females of *A. itadori* lay approximately 600–700 eggs, and nymphs pass through five instars [15]. Development from eggs to adults takes 33 days at 23 °C [21]. Both nymphs and adults use piercing mouthparts to remove phloem sap, and adults overwinter in the bark of neighboring trees [22]. Previous experiments have examined the relationship between day length and the onset of diapause in late summer, and the resulting data have been used to model voltinism in *A. itadori* across its introduced range in North America [2]. We aimed to bolster existing growth chamber experiments by assessing aspects of *A. itadori* phenology, as well as potential mortality factors, in field conditions in Canada.

In an experiment conducted in Whitby, Ontario (43.9593624, −78.9432246), we used small outdoor containers to expose overwintered *A. itadori* to *F. japonica* foliage at staggered times throughout the spring. This experiment allowed us to assess the following: (1) When and under what conditions do *A. itadori* become active in spring? (2) When and under what conditions do *A. itadori* begin laying eggs in spring? And finally, (3) how might the late emergence of knotweed foliage affect *A. itadori* survival?

## 2. Materials and Methods

### 2.1. Insect Rearing

All *A. itadori* used in these experiments were reared at Agriculture and Agri-Food Canada, Lethbridge Research and Development Centre (AAFC), Alberta, Canada. Psyllids from the Kyushu (southern) biotype were used, as these have been shown to perform better on *F. japonica* [16]. Our experiment used the population of the Kyushu biotype that was collected in 2016 by CABI UK (K2). Psyllids used in the experiment were reared on potted *F. japonica* plants and held at 15 °C and a 12 h/12 h light/dark cycle at AAFC for several weeks in early fall in order to stimulate the onset of diapause. Psyllids were then shipped to the University of Toronto on cut plants secured inside cardboard containers. On arrival, 100 psyllids were aspirated and sexed to determine an approximate sex ratio (42 males and 58 females). Psyllids were then placed into rearing cages prior to the initiation of the experiment. Psyllids were kept in mixed-sex populations prior to the onset of diapause, so they were likely mated prior to the start of the experiment.

### 2.2. Overwintering and Spring Emergence Experiment

**Figure 1:**
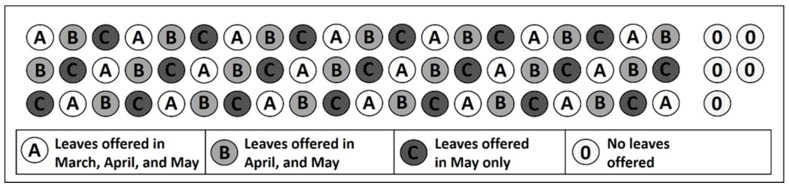
Allocation of treatment groups for experiment 1. Treatments were ordered systematically to control for any positional biases or variations in abiotic conditions within the experimental area. Five control containers, however, were placed immediately to the side of the main experimental grid to allow for early harvesting of adults without disturbing the main experiment.

Fifty *A. itadori* adults were placed inside each of 65 plastic overwintering containers and provided with *F. japonica* foliage at varying points throughout the spring in order to determine the timing of spring psyllid activity and oviposition and to observe *A. itadori* mortality during the transition between winter and spring. Fifty psyllids were used per container in order to capture natural variations in psyllid behavior. Sixty-five round 0.93 L plastic containers (height = 15.44 cm, diameter = 11.43 cm) (SC Johnson, Racine, WI, USA) were filled to 2 cm below the rim with a 50/50 mix of pine bark chips and playground mulch (Arnts the landscape supplier, Whitby, ON, Canada). On 13 November 2020, the 65 containers were buried up to 2 cm below the rim at a field site in Whitby, Ontario (43.9593624, −78.9432246). Sixty of the containers were buried in three rows of 20, with each container at least 5 cm apart, and a control group of five containers was buried 1 m away from the main experimental grid (Figure 1).

Finally, two additional containers were set up, without psyllids, at either end of the experimental grid. A LogTag temperature sensor (LogTag USA, Union, NJ, USA) was placed in both of these containers and programmed to record the temperature every hour throughout the winter and spring. Hourly temperature data were used to calculate cumulative degree days in the spring, using a minimum development threshold temperature of 6.9 °C [2].

Prior to their use in the experiment, a 5 cm diameter round hole was cut in the bottom of each container, along with two 2.5 cm diameter holes on either side. These holes were then covered with a fine plastic mesh (gauge < 1 mm) using a hot glue gun. The holes were intended to allow drainage and airflow between the containers and the surrounding soil, maintaining moisture levels in the containers approximately the same as the surrounding soil and leaf litter.

**Figure 2:**
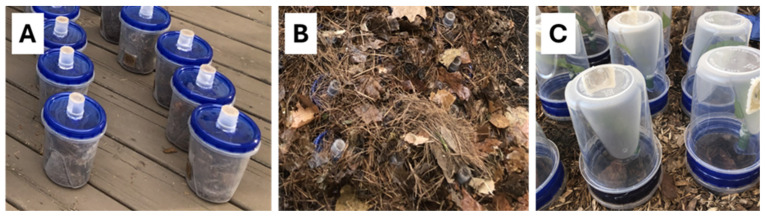
Experimental containers: (**A**) after psyllids had been added prior to burial in the fall; (**B**) after burial, such that only the snorkels were visible above the leaf litter; and (**C**) during observation periods during the spring when leaf material was made available to the psyllids.

**Figure 3:**
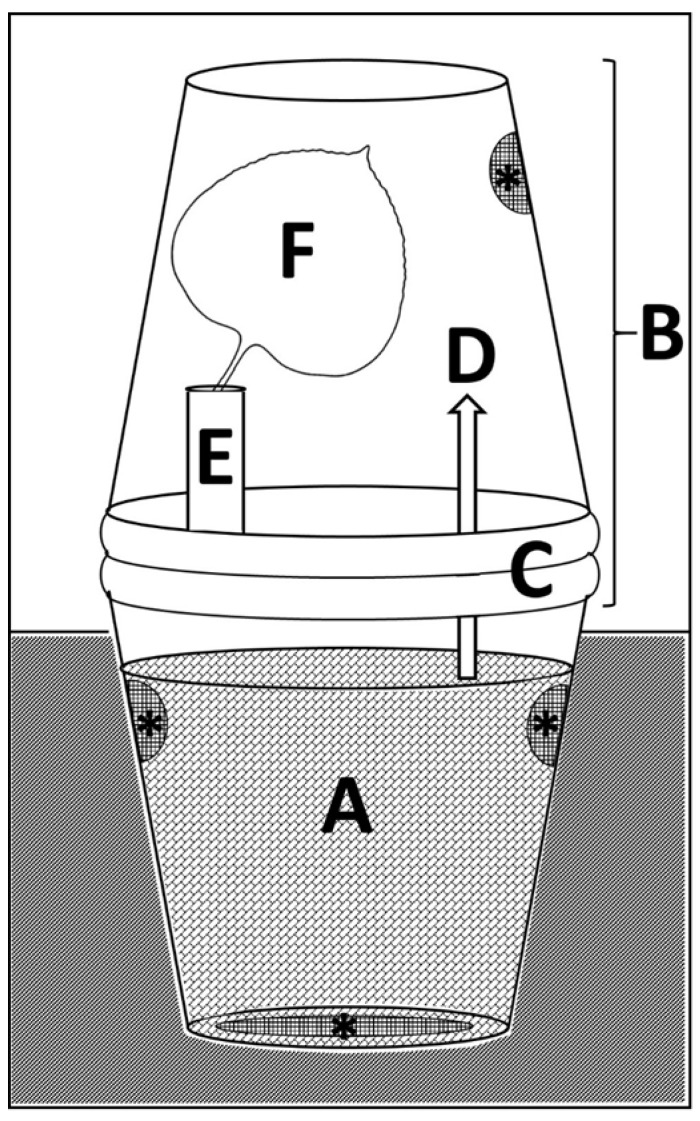
Experimental container design for psyllid monitoring. Overwintering containers were filled with a 50/50 mix of pine bark chips and playground mulch (**A**). During monitoring periods, the lids of overwintering containers were replaced with an overturned container (**B**) secured by two container lids glued end to end (**C**). The flat surface of those lids was removed to allow the psyllids free movement between the containers (**D**), with the exception of a small section that was used to attach a vial of florists’ foam (**E**). A sentinel leaf (**F**) was placed in the florist foam and held in place using parafilm. Gridded sections marked with * represent ventilation holes.

*Aphalara itadori* were aspirated from rearing cages into 50 mL centrifuge tubes. Tubes containing 50 psyllids were placed in a freezer at −18 °C for 30 s to reduce their mobility. Psyllids (50) were then added to each buried plastic container, and container lids were quickly screwed into place. Psyllids were not sexed prior to their addition to experimental cages; however, a roughly even sex ratio was assumed based on an overall assessment of the population (see above). Container lids were adapted by creating a 2.5 cm hole in the center using a Dremel tool and gluing a 25.47 mL plastic vial (Thermo Fisher Scientific, Waltham, MA, USA) over the hole, open side down. Another 2.5 cm hole was created in the bottom of the vial, which was then covered in a fine plastic mesh, as above, to create a snorkel (Figure 2A). After the container lids were in place, the grid of containers was buried in a mixture of pine bark and playground mulch so that only the snorkels were visible (Figure 2B). All experimental containers remained buried in this way throughout the winter, until psyllid monitoring commenced on 23 March. Spring psyllid activity was monitored in the experimental containers during three-day monitoring periods in March, April, and May. To monitor psyllid activity, soil and leaf litter were removed to expose the container lids. Container lids were removed and replaced with an inverted 0.93 L plastic container (Figure 2C). Any active psyllids were able to be viewed through this transparent container. Supported within the inverted containers was a single *F. japonica* leaf in florist foam (Oasis Floral Products, Kent, OH, USA) (Figure 3). The number of psyllids observed on these sentinel leaves was recorded over a period of three days during each monitoring period. Leaves were monitored at 10 am, 1 pm, and 4 pm on each day. To avoid double-counting the same psyllids, the maximum number of psyllids observed on the sentinel leaf at any one time was recorded as a measure of psyllid activity for each container.

The 65 containers were divided into three treatment groups (A, B, and C) based on the timing and frequency with which psyllid activity was monitored. Group A containers (n = 20) were monitored in March, April, and May. Group B containers (n = 20) were monitored in April and May. Group C containers (n = 20) were monitored in May only. The three monitoring periods began on 23 March, 18 April, and 14 May.

After each three-day monitoring period, *F. japonica* leaves were removed from the containers and searched under a dissecting microscope for any *A. itadori* eggs. Eggs on each leaf were counted, and any leaves bearing eggs were kept individually in freezer bags (17.7 cm by 18.8 cm) (SC Johnson, Racine, WI, USA). Eggs were monitored daily for hatching in order to confirm viability. The earliest appearance of viable eggs was recorded as a measure of how early in the season oviposition could commence, given the availability of foliage.

At the conclusion of the final foliage exposure on 16 May 2021, the number of surviving psyllids in each container was assessed. Any active psyllids were aspirated from each container. Leaves were then replaced in each container every two days, and active psyllids were removed periodically, keeping a record of the total number of psyllids recovered from each container. This process was repeated until two days passed without any further psyllid activity being observed. The number of surviving psyllids in the five control containers was determined using the same process, beginning on 23 March 2021. Psyllids were counted and aspirated from control containers starting in late March so that the number of surviving psyllids could be assessed prior to any spring mortality. Leaves were replaced every two days and searched daily for psyllid activity until a period of 1 week had passed without any further psyllid activity being observed.

### 2.3. Statistical Analysis

Psyllid activity was compared among monitoring periods (March, April, and May) using a generalized linear model with a negative binomial distribution and a log link function. The model had one independent variable (monitoring period) with three levels (March, April, and May) and one dependent variable, which was psyllid counts (the number of psyllids observed in containers during the monitoring periods). We included only the first observation period from each treatment group (A, B, and C) such that psyllid activity was not affected by previous foliage exposure and replication was equal among treatment groups (n = 20). Insect count data were tested for overdispersion and were not overdispersed (χ^2^/df = 0.35). Pairwise comparisons between monitoring periods were made using Wald χ^2^ comparisons with Bonferroni-adjusted *p* values.

To explore the effects of early spring feeding on psyllid survival to late spring, psyllid activity was compared among treatment groups (A, B, and C) during the May observation period using a generalized linear model with a negative binomial distribution and log link function. The model had one independent variable (prior feeding) with three levels (two, one, and zero prior exposures to spring foliage) and one dependent variable, which was psyllid counts (the number of psyllids observed in containers during the monitoring periods). Insect count data were tested for overdispersion and were not overdispersed (χ^2^/df = 0.36). Pairwise comparisons between monitoring periods were made using Wald χ^2^ comparisons with Bonferroni-adjusted *p* values. During the May observation period, containers in treatment groups A, B, and C had been given access to foliage twice previously, once previously, and never before, respectively.

After the final observation period in May, all living psyllids were harvested from the experimental containers. The number of psyllids was compared among treatment groups (A, B, and C) and control containers using a generalized linear model with a negative binomial distribution and a log link function. The model had one independent variable (treatment group) with four levels (treatment groups A, B, and C, and control containers) and one dependent variable, which was psyllid counts (the number of psyllids observed in containers during the monitoring periods). Insect count data were tested for overdispersion and were not overdispersed (χ^2^/df = 0.37). Pairwise comparisons between monitoring periods were made using Wald χ^2^ comparisons with Bonferroni-adjusted *p* values. All analyses were performed using SPSS version 29 (IBM, Armonk, NY, USA).

## 3. Results

**Figure 4:**
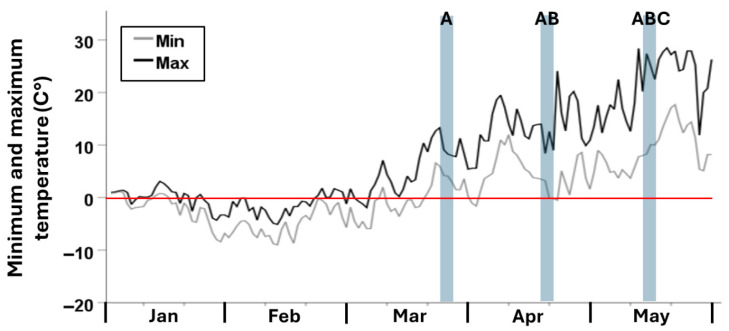
Minimum and maximum temperatures averaged from two LogTag sensors inside experimental containers. The red line highlights freezing events by marking 0 °C. Transparent blue bars represent the three periods during which psyllid activity was monitored in the containers. Letters on the blue bars indicate the treatment groups that were monitored during these periods.

The average winter temperature in the experimental containers, between 1 January and 22 March 2021, was −1.5 °C, and the minimum temperature was −9 °C. During March, April, and May psyllid observations, mean (range) temperatures were 8.6 °C (2.4–13.3 °C), 7.7 °C (3.2–14 °C), and 14.9 °C (8–27.4 °C), respectively. Between the three observation periods, there were two freezing events during which minimum temperatures dropped below 0 °C for two or more consecutive days. These events occurred beginning on 2 April and 21 April (Figure 4).

*Aphalara itadori* were observed to be active in very early spring, with the first observation of a psyllid on a sentinel leaf occurring on 23 March 2021. The earliest observation of oviposition occurred between 18 and 20 April 2021, and the eggs were confirmed to be viable.

**Figure 5:**
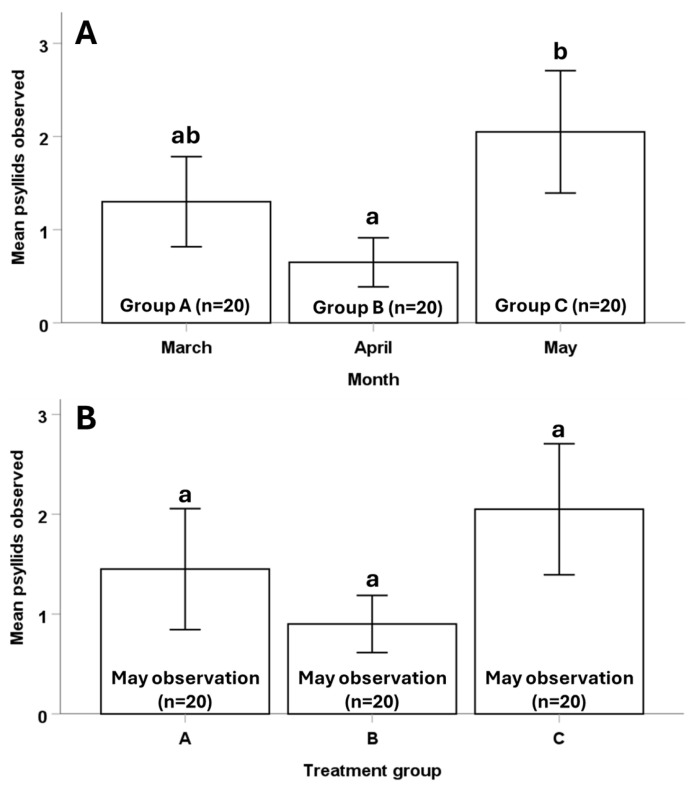
Comparison of psyllid numbers observed on sentinel leaves among (**A**) treatment groups A, B, and C during the March, April, and May monitoring periods, respectively, and (**B**) treatment groups A, B, and C during the May monitoring period. The data in panel (**A**) allowed us to compare psyllid activity among monitoring periods without any effects of prior foliage exposure. Each month, we monitored 20 experimental containers that had not previously been offered foliage. The data in panel (**B**) allowed us to assess the effects of prior exposure to foliage on psyllid activity in late spring because, during the May monitoring period, psyllids in treatment groups A, B, and C had previously been offered foliage twice, once, and zero times, respectively. Replicate sizes (numbers of experimental containers) are the same across all bars (n = 20). Error bars indicate standard error. Different lowercase letters indicate significant differences (α = 0.05).

Psyllid activity varied significantly among observation periods (March, April, May) (N = 60, χ^2^_2_ = 6.561, *p* = 0.038). Psyllid activity was significantly lower in April (mean ± SE: 0.65 ± 0.13) than it was in May (2.05 ± 0.32) (N = 40, *p* = 0.021). There was no difference in psyllid activity between March (1.3 ± 0.24) and April (N = 40, *p* = 0.149) or between March and May (N = 40, *p* = 0.270) (Figure 5A).

During the late spring (May) observation period, prior access to foliage did not affect the numbers of psyllids observed (N = 60, χ^2^_2_ = 3.766, *p* = 0.152). No differences in psyllid activity were observed between treatment group A (two prior exposures to foliage) and treatment group C (no prior exposure to foliage) (N = 40, *p* = 0.391), or between treatment group A and treatment group B (one prior exposure to foliage) (N = 40, *p* = 0.284). No differences in psyllid activity were observed between treatment groups B and C (N = 40, *p* = 0.068) (Figure 5B).

**Figure 6:**
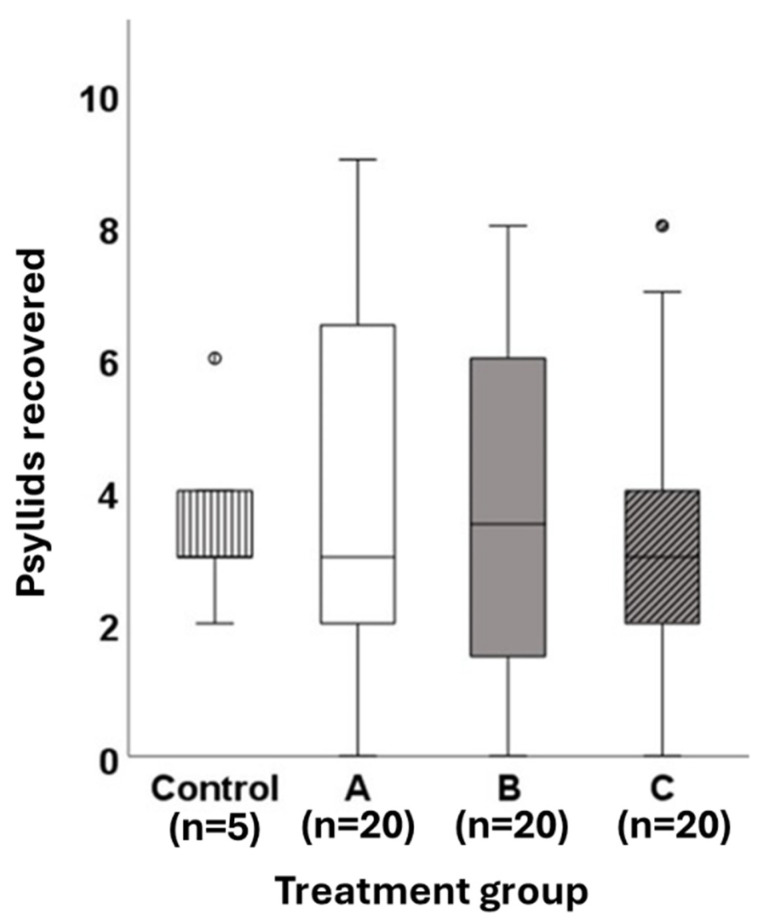
Surviving *A. itadori* adults recovered from control containers (n = 5) in March and treatment containers in May. *Fallopia japonica* foliage was placed in experimental cages, and active psyllids were removed using aspirators and counted. Treatment A containers (n = 20) had been provided with *F. japonica* leaves, and their activity was monitored visually in March, April, and May 2021. Treatment B containers (n = 20) were provided with *F. japonica* leaves, and their activity was monitored visually in April and May 2021. Treatment C containers (n = 20) were provided with *F. japonica* leaves, and their activity was monitored in May 2021 only. Box plots represent a summary of the data (minimum, first quartile, median, third quartile, and maximum). Open circles represent outliers (1.5× interquartile range (IQR)).

The number of psyllids recovered from containers at the end of the experiment was low across all treatments (mean ± SE: 3 ± 0.3), with approximately 7% of psyllids recovered overall. Total psyllid recovery did not differ among treatment groups or control containers (N = 65, χ^2^_3_ = 0.527, and *p* = 0.913) (Figure 6).

## 4. Discussion

*Aphalara itadori* were observed to be active in very early spring, with the first observation of a psyllid on a sentinel leaf occurring on 23 March 2021. The earliest viable eggs were observed between 18 and 20 April 2021. When the first viable eggs were laid, the day length at the experimental site was roughly 13:36 h (calculated using the method of Forsythe et al. [23] and including 1.5° of twilight), the maximum daily temperature (between 18 and 20 April) was 14 °C, and the post-diapause degree day accumulation was approximately 45 (based on a lower development threshold of 6.9 °C). Growth chamber experiments have previously identified similar oviposition thresholds. Grevstad et al. [2] found that 50% of *A. itadori* adults (Kyushu strain) cease reproduction in the fall at a day length of 14.1 h. Myint et al. [1] observed a lower threshold temperature for reproduction of ~15 °C, and a pre-oviposition period of ~4.5 days in optimal conditions (20 °C), which represented a predicted degree day accumulation of approximately 70. Our results are novel because they were obtained in Canadian field conditions. The results support the accuracy of earlier laboratory studies [1,2] but suggest that spring oviposition may occur somewhat earlier, at slightly lower temperatures and shorter day lengths, than previously predicted. Our results suggest that, in Canada, the conditions required for spring activity and oviposition in *A. itadori* will be met before the emergence of invasive knotweeds. The timing of the first spring generations of *A. itadori* in Canada will, therefore, be dictated by the phenology of local knotweed populations, rather than by the biology of the insects.

Overall psyllid activity across all treatment groups was significantly affected by observation month. This effect was due to a significant reduction in psyllid activity in April compared to later observations in May. Low overall activity in April may be explained by unseasonably low temperatures. Mean/minimum temperatures in containers during the April observations were 7.7/3.2 °C, compared to 8.6/2.4 °C and 14.9/8 °C, respectively. The first oviposition was observed during the April monitoring period, despite higher mean temperatures during the March observations. This suggests that oviposition was limited during the March monitoring period either by short day lengths or a lack of accumulated degree days, as predicted by previous laboratory studies [1,2].

In May, during the final monitoring period, *A. itadori* activity was not affected by the treatment group. These results confirm that psyllids are able to survive through to late May without access to food and support the conclusion of Clewley and Wright [24] that winter host feeding is not essential for successful overwintering in *A. itadori*. Additionally, this result suggests that psyllids can survive freezing events after feeding in early spring. For some freeze-avoidant insects, emptying the stomach of food helps to prevent ice formation and lower the supercooling point [25,26]. Although our results suggest that intermittent feeding did not prevent *A. itadori* adults from surviving subsequent periods of cold (temperatures dropped below freezing on four occasions after the first set of observations in March), high overall psyllid mortality made it impossible to characterize the relationship between psyllid feeding in early spring and subsequent survival.

At the end of the experiment, the number of surviving psyllids did not differ among the three treatment groups or the control group, in which survivorship was measured in March. These results suggest that *A. itadori* survivorship over the spring is high and that late emergence of knotweed in its introduced range is unlikely to cause significant mortality in the biological control agent. These results, again, should be treated with caution due to the high overall psyllid mortality observed in our experimental containers. The number of psyllids recovered from experimental containers was low across all treatments. This low recovery rate was surprising because laboratory populations of *A. itadori* in Canada have previously been overwintered using a similar methodology with high levels of success (Bourchier, unpublished data). The minimum winter temperature observed in the experimental containers was −9 °C. This represents fairly typical conditions for the region. Mean minimum winter soil temperatures (at a depth of 2.5 cm) between 2019 and 2021 were approximately −10 °C (NicheMapR version 3.3.2 [27]). It is possible that the small overwintering containers may have trapped excessive moisture, which could have affected the psyllids directly or impacted their ability to survive extreme cold [28]. Future research should explore the effects of soil moisture on the winter cold tolerance of *A. itadori*. An alternative explanation for the low survival of psyllids in our experiment is that spring temperatures inside the containers may have been too high. Although the maximum temperature recorded inside the containers was 28.5 °C, which is below the estimated maximum development threshold for the species [2], it is possible that the clear inverted containers could have trapped heat and elevated temperatures during the monitoring periods. Finally, it is possible that the small experimental containers did not provide enough suitable overwintering sites for the psyllids. A lack of deep bark crevices is thought to have contributed to low overwinter survival of *A. itadori* in a previous container study in the UK [24]. Future overwintering studies should be conducted in wider containers to ensure that ample overwintering locations at optimum litter depths are available to the psyllids. Future work should seek to explore the effects of abiotic factors such as humidity, soil moisture, and temperature fluctuations on the overwinter survival of *A. itadori*.

Voltinism models for *A. itadori* are currently based on temperature development data and knowledge of photoperiod-induced diapause [2]. These models can be enhanced by this study through the incorporation of knowledge about the timing of spring activity and oviposition. Predicting the number of generations a biological control insect will undergo in any part of its introduced range is important because it affects the rate of population increase as well as the degree of impact the agent may have on the weed. Understanding the duration of the reproductive season for a biological control agent in its introduced range can offer insights into the number of generations it can complete and highlight phenological or climatic asynchronies that might limit agent establishment [2,29]. In addition to supporting voltinism models for *A. itadori*, our results will inform the release program, indicating that fall releases of diapausing adults can be conducted throughout the introduced range, regardless of the timing of knotweed emergence. The observed robustness of *A. itadori* adults during spring provides support for the continuation of fall releases, which select for individuals that are able to survive the cold Canadian winters and ensure that psyllids have access to tender new shoots in the spring.

## Data Availability

The data that support the findings of this study are available at https://osf.io/wvgax/files/wfr9k, accessed on 20 December 2025.
